# Supplementary material for: Distinct patterns of incidental exposure to and active selection of radicalizing information indicate varying levels of support for violent extremism
Source: PLoS One. 2024 Feb 14;19(2):e0293810. doi: 10.1371/journal.pone.0293810 (PMC10866479; doi:10.1371/journal.pone.0293810)
Supplement: Attachment — Submitted filename: Reviewer responses PLOS One.docx [file pone.0293810.s001.docx]

**Responses to Reviewer Comments**

We thank all reviewers for their thoughtful comments, which have helped to improve the quality of the manuscript. In addition to the changes detailed below, we have revised the manuscript to improve readability and style.

**Reviewer 1**

1) Reviewer 1 noted that the sample should be described in more detail.

We agree that sample descriptions should always be as comprehensive as possible. We, therefore, had included in the original paper information about participants’ age, gender, and ethnicity. We had also directed readers to the Supplementary Material (specifically S1), which is available through the link to the OSF project page (shared before the sample description), where more information about participants’ level of education, work status, religion, citizenship, and immigration status is presented. We believe that the aforementioned information provides a detailed description of the sample.

**Reviewer 2**

1) Reviewer 2 highlighted that practical implications of the results are not discussed in sufficient detail. They asked us to address results in the context of UK PCVE efforts.

We agree that it is crucial to discuss practical implications of the findings. Although the data were collected in the UK, we aimed to provide broader points of discussion and, thus, did not focus on the UK explicitly. We added the following two paragraphs to explore implications for risk assessment and PCVE efforts that are implemented online, specifically redirection projects.

“Any practical implications that are based on one study or analysis can only be preliminary. We nevertheless believe that the results inform two fields of practice. First, we contribute insights to the development of individual risk assessment tools that aim to identify those vulnerable to violent extremism before they commit violence (Pressman & Davis, 2022). These tools (e.g., VERA(2) (Pressman & Flockton, 2012); TRAP-18 (Meloy et al., 2021)) typically include a wide variety of risk or protective factors, such as grievances and ideological commitment, criminal history, mental health, or the presence of a social support system. Previous research has further developed approaches to assess individuals’ risk based on textual digital trace data (i.e., linguistic risk assessment; Ebner et al., 2012; Cohen et al., 2014). Our findings suggest that information use behavior, which is to some extent observable, should be considered as well, and in a more nuanced way, in individual risk assessment and management. Namely, we showed that evidence of patterns of only incidental exposure to radicalizing material might be less of a concern, especially if it remains temporary (i.e., individuals are not likely to be embedded in environments where radicalizing messages are regularly disseminated). In turn, information use behavior defined by even a low probability of active selection of radicalizing content online or offline likely indicates stronger support for violent extremism which could manifest in acts of terrorism. Here, efforts to prevent radicalization are especially pertinent.

Perhaps somewhat ironically, in online settings, the fact that individuals actively seek radicalizing information could provide an avenue for targeted prevention. Notably, redirect(ion) projects, which have been implemented in collaboration with large social media platforms, use the search of keywords that are thought to be indicative of (violent) extremist or terrorist actors and narratives as a cue to provide counter-narratives or offer support by civil society organizations specialized in disengagement (Saltman et al., 2021). Evaluations of past programs highlight that their implementation is not without challenges (Helmus & Klein, 2018; Moonshot CVE, 2020). However, our results indicate that using the active search for radicalizing content as one of several indicators of risk is highly promising.” p. 21-22

2) Reviewer 2 also remarked that it would be helpful to introduce other theoretical frameworks (rather than the uses and gratification approach) to provide a rationale for the active selection of belief-congruent information.

We thank the reviewer for pointing us to those theories that were not yet familiar with. We removed the reference to uses and gratification theories and added a concise rationale grounded in the psychological immune system approach and Meaning Maintenance Model.

“In addition to being incidentally exposed, individuals also actively seek out content, sources, and media. Substantial evidence documents a general preference for information that aligns with existing beliefs and convictions (dissonance theory; Festinger, 1962). One reason for the active selection of belief-congruent information is the defense of one’s sense of self (Porot & Mandelbaum, 2021) and avoidance of threats to one’s self-image (psychological immune system; Gilbert, et al., 1998). The Meaning Maintenance Model (Heine et al., 2006) further stipulates that individuals “are inexhaustible meaning makers” (p. 91) and strive to keep previously established meaning frameworks intact; actively selecting information that confirms one’s beliefs might serve as a strategy to achieve this.” (p. 6)

3) Additionally, Reviewer 2 pointed out that the results for the additional measures of support for violent extremism were not available in the supplementary material.

We believe that this might have been a misunderstanding. The file “S2 Between-group Comparisons for Additional Measures for Violent Extremism.docx” in the folder ‘Supplementary Material’ on the OSF project page includes precisely the information that the reviewer had hoped to find (see <https://osf.io/zb2ux>).

4) Further, Reviewer 2 asked us to provide more information about the platform that we used for data collection, especially what data quality can be expected.

Responding to this comment, we added a reference to a recent paper that described the use of platforms such as Prolific for the study of violent extremism. Additionally, we added a sentence highlighting that we chose Prolific because it is expected to provide high-quality data.

“Data were collected in September 2021 on Prolific Academic, an online opt-in access panel (Clemmow et al., 2023b). We choose Prolific as a platform for recruiting participants because it has been consistently found to provide high(er)-quality data, especially as compared to other providers such as Amazon Mechanical Turk and CloudResearch (Douglas et al., 2023; Eyal et al., 2022).” (p. 10-11)

5) Lastly, Reviewer 2 made us aware of a typo on page 21 (now page 19), which we corrected.

**Reviewer 3**

1) The reviewer pointed out that no references were included in the abstract, and they suggested useful works to be added.

References were not included in the abstract to comply with the journal formatting requirements. On page 3, where the same argument is made again, we do provide several references (Bouhana & Wikström, 2010; Clemmow et al., 2022; Clemmow et al., 2023a; Gill et al., 2015; Hassan et al., 2018; Pauwels & Hardyns, 2018; Pauwels & Heylen, 2020; Pauwels & Schils, 2016; Perry et al., 2018; Rushchenko, 2019; Schils & Pauwels, 2016; Trujillo et al., 2009). We added to this list on page 3 the two recommended works.

2) Reviewer 3 further noted that the definition of radicalization that we provided is too narrow as it suggests that the latter is a natural path towards violence.

We adjusted the wording of the definition and included the recommended book as a reference.

“Radicalization refers to a process during which individuals adopt extremist attitudes and, in some but not all instances, “come to perceive acts of terrorism as a possible alternative for action” (Wikström & Bouhana, 2016, p. 176; see also Orofino & Allcorn, 2023).” p. 2

3) Further, reviewer 3 highlighted that it would be useful to replicate the study while focusing on specific ideologies.

We agree with this observation, although it is not something that can be achieved in the present research. We extended the call for replications in the discussion by adding a note on ‘different ideologies’:

“Going forward, the patterns that we have identified should be replicated across specific types of radicalizing content and ideologies.” p. 23

4) Reviewer 3 also reminded us that the study should be ideally replicated in a sample that is known to have adopted extremist beliefs to such a degree that it warrants support by a de-radicalization program.

We fully agree with this recommendation and extended the suggestions for future research in the following way: “Lastly, it is recommended to explore the research questions with other samples, notably, populations that have adopted (violent) extremist beliefs more strongly than the general population (e.g., individuals who are part of a de-radicalization program) or individuals who have committed or prepared acts of terrorism. In those instances, we would expect the absence of information use patterns that are defined by a near zero probability of incidental exposure to and active selection of radicalizing information or only evidence of incidental exposure.” p. 23

5) Lastly, Reviewer 3 highlighted that the findings might suggest that a higher likelihood of active selection of radicalizing information predicts the dissemination of the latter in other (online and offline) contexts.

We believe that this conclusion is not fully supported by the data, and we are, therefore, hesitant to make it (or add it to the manuscript). We do not have data on what participants do with the information that they actively select; future research could, of course, add such questions.

**Reviewer 4**

1) Reviewer 4 noted that it is crucial to document that the concepts of IS, AS, as well as the dependent variables are distinct. They recommended that we conduct a factor analysis to confirm that the measured concepts do not ‘overlap’.

We agree with the reviewer and thank them for raising the point. We report an exploratory factor analysis with oblimin rotation in the Supplementary Material (S5) that documents that the scales capturing active selection and incidental exposure assess distinct concepts and that both are distinct from the concept examined with the outcome measures – support for violent extremism. The exploratory factor analysis also shows that all items of the radicalism intentions sub-scale and the violent extremist attitudes scale loaded on one factor, which explains why results for both measures are highly similar. We added a note indicating this point in the manuscript (with a reference to Supplementary Material S5).

“The highly similar result pattern for both outcome variables is not surprising; indeed, an exploratory factor analysis with oblimin rotation showed that all items measuring the two dependent measures loaded on one factor (Supplementary Material S5).” p. 18
